# The Absence of the N-acyl-homoserine-lactone Autoinducer Synthase Genes *traI* and *ngrI* Increases the Copy Number of the Symbiotic Plasmid in *Sinorhizobium fredii* NGR234

**DOI:** 10.3389/fmicb.2016.01858

**Published:** 2016-11-18

**Authors:** Jessica Grote, Dagmar Krysciak, Katrin Petersen, Simon Güllert, Christel Schmeisser, Konrad U. Förstner, Hari B. Krishnan, Harald Schwalbe, Nina Kubatova, Wolfgang R. Streit

**Affiliations:** ^1^Department of Microbiology and Biotechnology, Biocenter Klein Flottbek, University of HamburgHamburg, Germany; ^2^Core Unit Systems Medicine, University of WürzburgWürzburg, Germany; ^3^Plant Genetics Research Unit, Agricultural Research Service, United States Department of Agriculture, University of MissouriColumbia, MO, USA; ^4^Institute for Organic Chemistry and Chemical Biology, Center for Biomolecular Magnetic Resonance Johann Wolfgang Goethe-UniversityFrankfurt, Germany

**Keywords:** *Sinorhizobium fredii*, plasmid copy number, plant symbioses, quorum sensing (QS), RNA sequencing (RNA-Seq)

## Abstract

Plant-released flavonoids induce the transcription of symbiotic genes in rhizobia and one of the first bacterial responses is the synthesis of so called Nod factors. They are responsible for the initial root hair curling during onset of root nodule development. This signal exchange is believed to be essential for initiating the plant symbiosis with rhizobia affiliated with the *Alphaproteobacteria*. Here, we provide evidence that in the broad host range strain *Sinorhizobium fredii* NGR234 the complete lack of quorum sensing molecules results in an elevated copy number of its symbiotic plasmid (pNGR234*a*). This in turn triggers the expression of symbiotic genes and the production of Nod factors in the absence of plant signals. Therefore, increasing the copy number of specific plasmids could be a widespread mechanism of specialized bacterial populations to bridge gaps in signaling cascades.

## Introduction

The rhizobium–legume symbiosis is considered to be one of the best-studied model systems of mutualistic interactions between eukaryotic hosts and the *Alpha*- and *Betaproteobacteria* that are commonly called “rhizobia.” The symbiosis in the *Alphaproteobacteria* is initiated by a signal exchange between the legume plant and the microbe that ideally results in the production of nitrogen-fixing plant root nodules (Gage, 2004; Jones et al., 2007; Deakin and Broughton, 2009). The infection depends in part on the release of plant-produced specific *nod* gene-inducing flavonoids (Spaink, 2000; Gage, 2004; Mierziak et al., 2014; Nelson and Sadowsky, 2015). These polyphenolic compounds are perceived by bacterial sensors, i.e., NodD, that induce expression of genes responsible for the synthesis of Nod factors. The Nod factors are afterwards released by the bacteria and trigger the nodulation pathway in susceptible legumes. Then, the rhizobia enter the root tissue through infection threads in the root hairs, find their way into the cortex, multiply and colonize the intracellular spaces in root nodules (Gage, 2004; Oldroyd, 2013). While most rhizobia and closely related species are known to establish a symbiosis with a rather small number of plant legume genera, some rhizobia have evolved mechanisms that allow them to nodulate a larger variety of legume plants. These strains have been designated “broad host range” strains and are promiscuous with respect to the selection of their host plants (Broughton and Perret, 1999; Krysciak et al., 2015). *Sinorhizobium fredii* NGR234 (hereafter NGR234) is well known for its exceptional wide host range with more than 120 different genera of legumes and the non-legume *Parasponia andersonii* (Broughton and Perret, 1999; Pueppke and Broughton, 1999). The NGR234 tripartite genome encodes for two *N*-acyl-homoserine-lactone (HSL) autoinducer (AI) synthases from the LuxI family, designated *traI* and *ngrI*. The *ngrI* gene is encoded on the bacterial chromosome and *traI* is located on the symbiotic plasmid pNGR234*a*. The *traI* gene is part of a conserved cluster that shares a high degree of synteny with the well-studied tumor inducing (Ti) plasmid of *Agrobacterium tumefaciens* (He et al., 2003; Schmeisser et al., 2009; Pinto et al., 2012; Krysciak et al., 2014). pNGR234*a* is a *repABC*-type plasmid similar to the *A. tumefaciens* Ti plasmids and other known rhizobial symbiotic plasmids (Cevallos et al., 2008; Pinto et al., 2012).

Previously, it was reported that in *A. tumefaciens* the copy number of the Ti plasmid was controlled in an AI-dependent manner and that tumorigenesis was increased by the presence of AI molecules (Pappas, 2008; Pinto et al., 2012). Because of these reports and the high synteny between the *traI*-*repA* intergenic regions of *A. tumefaciens* and NGR234, we asked if the copy number of pNGR234*a* is controlled in a similar way and if this could affect the symbiotic interaction with the plant. Within this manuscript, we provide evidence that NGR234 has evolved a previously unrecognized mechanism that increases copy number of pNGR234*a*. This is, in contrast to *A. tumefaciens*, achieved by the absence of AI molecules. Thereby, the symbiotic genes are activated in a previously not known and flavonoid-independent manner. This strategy may be another key to the broad host range phenomenon of NGR234.

## Materials and methods

### Bacterial strains, growth conditions, and measurement

Bacterial strains and plasmids used in this work are listed in Supplementary Table 1. *Sinorhizobium fredii* NGR234 was routinely grown at 30°C in liquid TY medium (0.5% tryptone, 0.25% yeast extract, 10 mM CaCl_2_, pH 7.0) or YEM medium (55 mM mannitol, 0.1% yeast extract, 3 mM K_2_HPO_4_, 0.8 mM MgSO_4_, 1.7 mM NaCl, 0.013 mM CaCl_2_, and 0.14 mM FeCl_3_) at 160 rpm. *Escherichia coli* was grown in LB medium at 37°C and supplemented with the appropriate antibiotics (Sambrook and Russell, 2001). The OD_600_ was measured at indicated time points.

### Construction of *Sinorhizobium fredii* NGR234 deletion mutants

Molecular cloning steps were in general done as outlined in reference (Sambrook and Russell, 2001). For the construction of a deletion of the *traR* gene a 650 bp PCR fragment containing the 249 bp upstream region of *traR* and the 399 bp downstream fragment flanking the *traR* gene were cloned in the suicide vector pNPTS138-R6KT (Lassak et al., 2010). For the deletion of the *traM* gene, a flanking region of 485 bp upstream and 744 bp downstream were amplified, ligated and cloned into pNPTS138-R6KT. PCR fragments were amplified using primers as indicated in Supplementary Table 2. The resulting constructs (pNPTS138-R6KT::Δ*traR* and pNPTS138-R6KT::Δ*traM*) were transformed into NGR234 by conjugation. Single recombinant clones carrying the construct were selected on TY medium containing kanamycin and rifampicin. To obtain double recombinant mutants, bacteria were cultured overnight in liquid TY medium lacking the antibiotics and plated on TY medium in the presence of 10% sucrose the next day for *sacB* counterselection. The deletion mutants were all verified by PCR analyses, sequencing of the complete genomes using Miseq technology and generating 4–5.5 million reads for each mutant.

### RNA extraction, library construction, sequencing, and bioinformatical analysis of transcriptome samples

For the NGR234 wild type and the double deletion mutant strain RNA-seq libraries were constructed from independent biological RNA samples in triplicate. Samples were harvested from stationary growth phase cultures (Supplementary Table 3). For the experiments, cells were grown for 48 h to a final OD_600_ not greater than 9.1 for the wt and 4.7 for the double deletion mutant prior to total RNA extraction (Supplementary Table 3). For all samples cDNA libraries were constructed and sequenced. For each of the treatments three independent biological experiments were performed and examined by RNA-seq. Total RNA extraction, RNA-sequencing and data analysis was done as recently published for NGR234 (Krysciak et al., 2014). Alignments were established and for each sample a min. of 0.5–2.2 million cDNA reads could be uniquely mapped to the NGR234 genome resulting in 3.9–5.2 million uniquely mapped reads per treatment (Supplementary Table 3). Genes with a fold-change of ≥ 2.0 and an adjusted *p*-value (*p*-value was corrected by FDR (false discovery rate) based on Benjamini-Hochberg procedure) of ≤ 0.05 were considered as differentially expressed. The raw, de-multiplexed reads as well as coverage files have been deposited in the National Center for Biotechnology Information's Gene Expression Omnibus under the accession number GSE78039. Differential RNA-seq data were depicted using the circos software 0.64 (Krzywinski et al., 2009). Detailed results are shown in Supplementary Table 4.

### Quantitative real time PCR (qRT-PCR) and quantitative PCR (qPCR)

QRT-PCR experiments were carried out to verify selected QS-regulated genes, as previously described (Krysciak et al., 2014). Gene-specific primers used for qRT-PCR are listed in Supplementary Table 1. The qRT-PCR reactions were set up according to manufacturer's protocol using the SYBR® Select Mater Mix for CFX (Applied biosystems® by life technologies, TX, USA) and performed with the CFX96 Touch™ Real-Time PCR Detection System (Bio-Rad Laboratories, Munich, Germany). To normalize variability in expression levels, *rpoD* and *recA* were used as the internal control genes. Data were analyzed based on the normalized gene expression [2^−ΔΔC(t)^ method] and the CFX Manager™ software (Release 3.1, Bio-Rad Laboratories, Munich, Germany).

To verify the copy number of pNGR234*a*, total DNA (genomic DNA, gDNA) was extracted of the corresponding *Sinorhizobium fredii* NGR234 culture under the conditions indicated in Tables 1, 2 with the peqGOLD Bacterial DNA Kit (PEQLAB Biotechnologie GmbH, Erlangen, Germany). Cells were in general cultivated for 48 h if not otherwise stated. The qPCR reactions were set up according to manufactures protocol using the SYBR® Select Mater Mix for CFX (Applied biosystems® by life technologies, TX, USA) and performed with the CFX96 Touch™ Real-Time PCR Detection System (Bio-Rad Laboratories, Munich, Germany). Standard curves of 10-fold serial dilutions of DNA were generated for each gene to evaluate the primer efficiency and for data analysis. The efficiency, slope and correlation coefficient were determined by the CFX Manager™ software (Release 3.1, Bio-Rad Laboratories, Munich, Germany). All qPCR and qRT-PCRs reactions were run in triplicate and repeated at least three times in separate experiments under the same conditions. To normalize variability in copy levels, NGR_c03800 and *recA* were used as the internal control genes for plasmid and chromosome in the qPCR experiments, respectively. For the spent culture experiments, the NGR234 wild type was grown for 48 h before centrifugation. Afterwards, the supernatant was sterile filtered and added to a newly inoculated NGR234-Δ*traI*-Δ*ngrI* culture (10% vol./vol.).

**Table 1:** **Copy number of pNGR234***a*** estimated using qPCR and primers for the ORFs NGR234_a00010 and NGR234_a01270**.

**Strain/Treatment**	**Copy number/target gene:**	
	**NGR_a00010**	**NGR_a01270**
NGR234 wt 24 h	1.1 ± 0.04	1.2 ± 0.04
NGR234 wt 48 h	1.0 ± 0.03	1.0 ± 0.05
NGR234 wt 72 h	1.4 ± 0.13	1.1 ± 0.11
NGR234 wt 96 h	0.9 ± 0.07	1.0 ± 0.06
NGR234-Δ*traI*-Δ*ngrI* 24 h	2.8 ± 0.22	2.9 ± 0.21
NGR234-Δ*traI*-Δ*ngrI* 48 h	6.2 ± 0.35	6.0 ± 0.28
NGR234-Δ*traI*-Δ*ngrI* 72 h	7.5 ± 0.25	5.6 ± 0.32
NGR234-Δ*traI*-Δ*ngrI* 96 h	8.7 ± 2.31	8.4 ± 2.25

*Results are mean values with the corresponding standard deviation of three technical replicates and of three independent biological samples. Cells were grown in YEM medium. The primer efficiencies were 90.1% for the NGR234_a00010 primers and 90.5% for the NGR234_a01270 primers. Copy numbers were calculated based on control qPCRs for chromosomal genes (NGR234_c03800 and NGR234_c16470) and in the background of the wild type*.

**Table 2:** **Copy number of pNGR234***a*** estimated using qPCR and primers for the ORFs NGR234_a00010 and NGR234_a01270**.

**Strain/Treatment**	**Copy number/target gene:**	
	**NGR_a00010**	**NGR_a01270**
NGR234	1.0 ± 0.3	1.0 ± 0.3
NGR234::pBBR1MCS-2::*traI*	0.9 ± 0.1	0.9 ± 0.1
NGR234-Δ*ngrI*	1.3 ± 0.1	1.5 ± 0.2
NGR234-Δ*ngrI*_c*ngrI*	1.9 ± 0.1	2.9 ± 0.2
NGR234-Δ*traM*	1.1 ± 0.1	1.5 ± 0.1
NGR234-Δ*traI*	1.5 ± 0.1	1.8 ± 0.1
NGR234-Δ*traI*_c*traI*	2.5 ± 0.2	3.4 ± 0.3
NGR234-Δ*traR*	1.1 ± 0.06	1.1 ± 0.04
NGR234-Δ*traI*-Δ*ngrI_*c*traI*c*ngrI*	2.0 ± 0.3	3.3 ± 0.3
NGR234-Δ*traI*-Δ*ngrI_*c*traI*	0.7 ± 0.2	0.6 ± 0.2
NGR234-Δ*traI*-Δ*ngrI_*c*ngrI*	0.9 ± 0.2	0.8 ± 0.2
NGR234 pBBR1MCS-2	0.7 ± 0.02	0.8 ± 0.01
NGR234 pBBR1MCS-2::*repA0*	3.1 ± 0.2	3.8 ± 0.3
NGR234 pBBR1MCS-2::*repX*	0.8 ± 0.1	1.0 ± 0.1

*Results are mean values with the corresponding standard deviation of three technical replicates and of three independent biological samples. Cells were grown for 48 h on YEM medium. Cells were assayed in early stationary phase. Sinorhizobium fredii NGR234 wild type cells had an OD of 9–10 and mutant cells an OD of 3–4. The primer efficiencies were 90.1% for the NGR234_a00010 primers and 90.5% for the NGR234_a01270 primers. Copy numbers were calculated based on control qPCRs for chromosomal genes (NGR234_c03800 and NGR234_c16470) and in the background of the wild type. cngrI, complementation construct of ΔngrI; ctraI, complementation construct of ΔtraI, ctraI-cngrI complementation construct for the ΔtraI-ΔngrI double deletion mutant*.

### SDS-page and western blot analysis

Extracellular proteins from rhizobia were obtained following the procedure described earlier (Lorio et al., 2010). Immunoblot analysis was performed using a cocktail of antibodies raised against the individual nodulation outer proteins (Nops) at a final dilution of 1:10,000. Immunoreactive proteins were detected with an enhanced chemiluminescent substrate (Super Signal West Pico kit; Pierce Biotechnology, Rockford, IL) according to the manufacturer's instructions.

### Root hair curling assays

For the root hair curling assay, supernatants of 48 h 500 ml cultures grown in TY medium of either uninduced or induced (1 μM apigenin) cultures of NGR234 or NGR234-Δ*traI*-Δ*ngrI* were collected. The Nod factors were extracted with 0.4 volumes of *n*-butanol according to (López-Lara et al., 1995). The butanol layer was collected; the butanol removed by rotary evaporation and the residue was resolved in water over night to a final volume of 1/100 of the starting culture.

To test the biological activity of the extracted Nod factors *Vigna unguiculata* seedlings were sterilized and germinated as previously published (Krysciak et al., 2011). Sterile germinated seedlings were transferred to glass containers supplemented with glass beads and Hoagland medium (Hoagland and Arnon, 1950). The seedlings were grown for additional 24 h under the following conditions: day/night; 24/19°C; 16/8 h; 60% relative humidity. The small plants were transferred into a small plastic container and coated with 500 μl Hoagland medium and 500 μl supernatant extract or 1 ml supernatant extract of the NGR234 cultures. After 24 h incubation in the dark, the root hairs were analyzed using a Zeiss AxioCam microscope with an MRm camera mounted on the microscope (Zeiss Axio Imager.M2). Images were recorded with a 40x magnification.

## Results

### NGR234 increases the pNGR234a copy number in the complete absence of homoserine-lactone-like (HSL) autoinducer (AI) molecules

**Figure 1:**
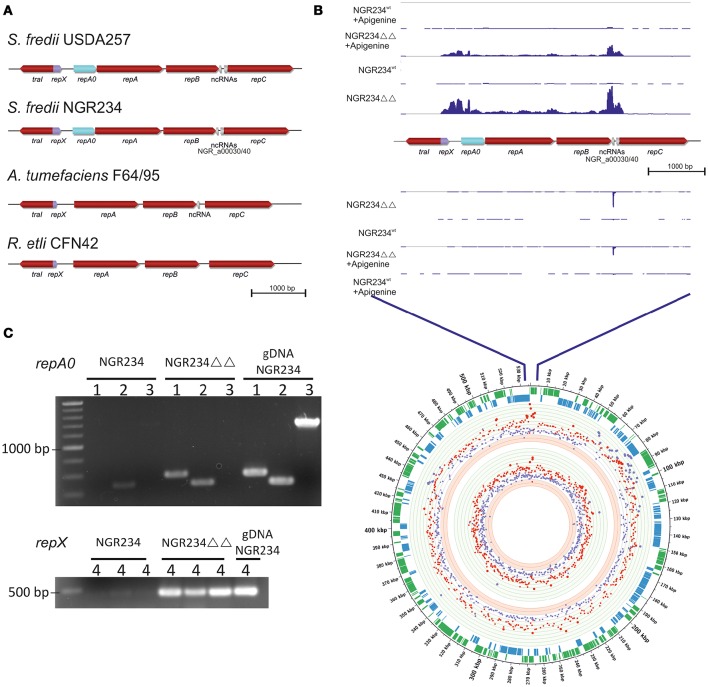
**Genomic organization and analyses of ***repX*** and ***repA0***. (A)** Genomic organization of the *traI* and *repABC* genes including the noncoding RNAs (NGR_a00030/40) and the two ORFs *repX* and *repA0* in the intergenic region between *traI* and *repA* of the two broad host range strains *Sinohrizobium fredii* NGR234 and USDA257 and two closely related strains *Agrobacterium tumefaciens* F64/95 and *Rhizobium etli* CNF42. Sequences were extracted from NCBI accession numbers NC_000914.2, NC_018000.1, NC_019555.1 and NC_007762.1 and compared to the GenBank entry BK009410. **(B)** The upper part of this picture represents the mapped transcripts on the genomic section around the origin of replication in induced (1 μM apigenin) and in uninduced NGR234-Δ*traI*-Δ*ngrI* and wild type cells. The image was composed using the integrated genome browser software version 8.4.4. Mapped transcripts above the annotated genes represent transcripts on the leading strand and mapped transcripts under the annotated genes represent transcripts on the lagging strand. The lower part of this figure section shows a circular representation of the RNA-seq-based transcriptome data for pNGR234*a* of the NGR234-Δ*traI*-Δ*ngrI* mutant vs. the wild type and the wild type strain induced with apigenin vs. the uninduced wild type. The circular diagram was calculated using the circos software 0.64 (Krzywinski et al., 2009). Fold change cut-off: log_2_ 8/−3 (circle size by values). The circle described from the outside to the innermost circle: Outer circle indicates coordinates of the pNGR234*a* in kbp. The second and third circles indicate the ORFs on the leading (green) and the lagging (blue) strand. The circles in light green indicate log_2_ 8; 7; 6; 5; 4; 3; 2, the white circles in-between the colored once represent log_2_ 1/−1 and the light red circles indicate log_2_ −2; −3 for the sense transcripts. The next circles represent the same log_2_ values for the antisense transcripts. The dots scattered over the light green, light red and white circles represent the transcripts of NGR234-Δ*traI*-Δ*ngrI* in comparison to the wild type strain (red) and the wild type strain induced with 1 μM apigenin in comparison to the wild type strain (purple). **(C)** End point RT-PCR of *repA0* and *repX* on cDNA generated of RNA from the wild type and NGR234-Δ*traI*-Δ*ngrI*. Samples are shown on a 0.8% agarose gel with a 1 kb λ marker (Thermo Scientific, Braunschweig, Germany). Primer pairs used for the amplification: 1, amplification of the *repA0* gene (parA_int_fw and parA_int_rev); 2, amplification of the *repA* gene (RT_repA_fw and RT_repA_ rev); 3, amplification of a possible elongated *repA* gene (parA_int_fw and RT_repA_ rev); 4, amplification of the *repX* gene in three independent biological samples (repX_int_fw and repX_int_rev).

The pNGR234*a* replicon belongs to the *repABC*-type plasmids and there is a high degree of synteny with respect to the overall organization of the region encoding the *traI* and the *repABC* genes with *A. tumefaciens* Ti plasmid and many other *repABC*-type plasmids (Table 3, Figure 1A). In *A. tumefaciens* the Ti plasmid copy number is increased in the presence of elevated levels of the AI molecule 3-oxo-C8-HSL and in *Rhizobium leguminosarum* the pRL1J *repA* transcription is increased in the presence of the same AI (Pappas and Winans, 2003a; McAnulla et al., 2007). Mainly because of these earlier reports we wondered if in NGR234 a similar effect on the copy number of pNGR234*a* or the *repA-E* gene transcription could be observed and if any of the genes involved in AI biosynthesis would be involved in this response. To address these questions, and to test the effect of the complete absence of HSL-like AI molecules we analyzed the copy numbers of the pNGR234*a* symbiotic plasmid using quantitative PCRs on the genomic DNA in the background of the parent strain and in the background of various NGR234 AI synthase mutants including a recently constructed Δ*traI*-Δ*ngrI* double deletion mutant. All mutants were either genetically or chemically complemented and the mutations verified by PCR or whole genome sequencing in this study or in a previous study (Krysciak et al., 2014). Thereby, it is notable that only the NGR234-Δ*traI*-Δ*ngrI* mutant grew significantly slower compared to its parent strain. It had doubling times of 5.9 vs. 3.6 h for the parent strain and it reached stationary phase almost at the same time of cultivation (after 35–40 h growth) as the parent strain at an OD_600nm_ of 3–5. However, the NGR234 parent usually grew to an OD_600nm_ of 9–10 (Supplementary Figure 1). The genetically complemented mutant carrying both *traI* and the *ngrI* genes on the broad host vector pBBR1MCS-2 was not affected in its growth (Supplementary Figure 1). This observation confirmed the correctness of the mutation and that no unspecific mutational events were the cause of the slow growth of the double deletion mutant.

**Table 3:** **Number of base pairs in the intergenic region of ***traI*** and ***repA*** in various rhizobial and agrobacterial species carrying ***repABC***-like plasmids**.

**Strain**	***traI-repA* (bp)**	**Strain**	***traI-repA* (bp)**	**Strain**	***traI-repA* (bp)**
***SINORHIZOBIUM***					
*S. fredii* NGR234	803	*R. sullae* WSM1592	372	***RHIZOBIUM TROPICI***	
*S. fredii* USDA257	803	*R. larrymoorei* ATCC 51759	367	*R. tropici* CIAT 899	376
*S. fredii* HH103	803	*R. rubi* NBRC13261	366	*R. tropici* USDA 9039	376
*S. fredii* GR4	383	*R. giardinii* bv. *giardinii* H152T	210	*R. tropici* USDA 9039	393
*S. fredii* GR64	442/382			*R. tropici* PRF 81	389
*S. terangae* WSM1721	354	***AGROBACTERIUM***		*R. tropici* CF286	376
*S. arboris* LMG1419	354	*A. tumefaciens* GW 4	377	*R. tropici* YR635	393
*S. americanum* CCGM7	382	*A. tumefaciens* A4	388		
*S. melilloti* GVPV12	442	*A. tumefaciens* 5A	490	***RHIZOBIUM LEGUMINOSARUM***	
*S. meliloti* Rm41	291	*A. tumefaciens* CCNWGS0286	367	*R. leguminosarum* bv. *trifolii* CC287f	360
*S. meliloti* 4H41	382	*A. tumefaciens* F2	239	*R. leguminosarum* bv. *trifolii* CC283b	1652
		*A. tumefaciens* LBA4404	365	*R. leguminosarum* bv. *trifolii* CB782	375
***RHIZOBIUM*****SP. AND OTHERS**		*A. tumefaciens* pTiBo542	365	*R. leguminosarum* bv. *trifolii* TA1	364
*Rhizobium* sp. OK665	366	*A. tumefaciens* LBA4213 (Ach5)	342	*R. leguminosarum* bv. *trifolii* WSM1325	364
*Rhizobium* sp. OR191	688	*A. tumefaciens str*. C58	363	*R. leguminosarum* bv. *viciae* 128C53	376
*Rhizobium* sp. YR295	376	*R. rhizogenes* A4	315	*R. leguminosarum* bv. *viciae* Vc2	376
*Rhizobium* sp. YR519	365	*R. rhizogenes* NBRC13257	441	*R. leguminosarum* bv. *viciae* UPM1131	376
*Rhizobium* sp. CF258	376	*R. rhizogenes* YR147	393	*R. leguminosarum* bv. *viciae* UPM1137	376
*Rhizobium* sp. CF080	351	*A. rhizogenes* ATCC 15384	388	*R. leguminosarum* bv. *viciae* 3841 (pRL7)	387
*Rhizobium* sp. BR816	383	*A. rhizogenes* plasmid pRi1724	316	*R. leguminosarum* bv. *viciae* 248	376
*Rhizobium* sp. CCGE 502	372	*A. rhizogenes* plasmid pRi2659	316	*R. leguminosarum* bv. *viciae* WSM1455	364
*Rhizobium* sp. STM6155	373	*Agrobacterium* sp. ATCC 31749	377	*R. leguminosarum* bv. *viciae* GB30	364
*N. galegae HAMBI 1141*	337	*Agrobacterium* sp. Cherry 2E 2–2	358	*R. leguminosarum* bv. *viciae* Ps8	364
*A. doebereinrae UFLA1-100*	577	*Agrobacterium vitis* S4 pTiS4	361	*R. leguminosarum* bv. *phaseoli* 4292	434
*M. tianhanense CGMCC 1.2546*	531	*A. albertimagni* AOL15	316		
*B. japonicum* USDA 135	531				
*B. japonicum* USDA 123	494	***RHIZOBIUM ETLI***			
*R. leucaenae* USDA 9039	376	*R. etli* bv. *mimosae* Mim1	162		
*R. lusitanum* P1-7	388	*R. etli* bv. *mimosae* IE4771	337		
*R. giardinii* bv. *giardinii* H152T	210	*R. etli* CFN 42 (p42d)	374		
*R. selenitireducens* BAA-1503	388	*R. etli* CFN 42 (p42a)	373		
*R. loessense* CGMCC 1.3401	356				
*R. mesoamericanum* STM 3625	388	***ENSIFER***			
*R. mongolense* USDA1844	304/374	*Ensifer* sp. USDA 6670	295		
*R. hainannese* CCBAU 57015	379	*Ensifer adhaerens* OV14	2683		

To assess the copy numbers, the different strains were grown in YEM medium, DNA was extracted and quantitative PCRs were performed as described in Material and Methods sections. In general, we observed that the copy number of pNGR234*a* in the parent strain was 1 copy per chromosome independent from the growth phase and culture age (Tables 1, 2 and Supplementary Figure 1). We monitored growth and copy numbers at 24, 48, 72 and 96 h for the wild type and the double deletion mutant. Interestingly, we observed that the copy number of pNGR234*a* in the double deletion mutant NGR234-Δ*traI*-Δ*ngrI* increased during growth in YEM medium. After 24 h the counts were almost three copies per cell and we measured six copies of pNGR234*a* after 48 h. After 96 h a copy number of eight was observed (Table 1).

To verify these findings, we initially attempted to complement the double deletion mutant chemically by adding small amounts of 3-oxo-C8-HSL to cultures. However, these tests were not conclusive as the solvent used for the AI molecule (DMSO) interfered with the copy numbers of pNGR234*a* in the wildtype strain as well. Therefore, we assayed the copy numbers of pNGR234*a* in the background of genetically complemented mutants carrying either the *traI* or the *ngrI* genes as single genes. In a further control we added both genes back into the double deletion mutant using the pBBR vector. Furthermore, we analyzed the copy numbers of pNGR234*a* in the background of the two single AI synthase mutants NGR234-Δ*traI* and NGR234-Δ*ngrI*, the corresponding complemented strains and a *traR* and a *traM* deletion mutant (Table 2). Finally, we added supernatant from a 48-h culture to fresh cultures from the double deletion mutant. In these tests, we could partially complement the double deletion mutant by reducing the copy number from 5.8 (±0.3) copies per cell to 4.3 (±0.5) copies per cell.

The copy number of pNGR234*a* was increased in the background of a mutant carrying a deletion in *traI*. In this mutant the copy number of pNGR234*a* was increased to 1.5–1.8 copies per cell after 48 h of growth (Table 2). The copy numbers of pNGR234*a* were found to be 1.9–3.4 copies per chromosome when extra copies of the *traI* and *ngrI* genes were introduced into the corresponding deletion mutant on a self-replicable plasmid (Table 2). The control experiments in which, we used the double deletion mutant carrying either the *traI* or the *ngrI* gene on the pBBR vector indicated that it was sufficient to introduce one of these genes to reduce the copy number back to wild type level (Table 2). Therefore, these findings imply that a cross-regulation between the TraI/R and the NgrI/R systems is likely and that the TraI/R system is perhaps more important since its deletion already leads to a slight upregulation of the plasmid copy number.

However, no significant increase in copy number was observed for strains carrying single deletions in *ngrI, traR* and *traM* (Table 2) and in the parent strain carrying extra copies of *traI* or *ngrI* (data not shown).

Altogether the results described above, imply that NGR234 increases the pNGR234*a* copy number in the absence of 3-oxo-C8-HSL molecules (double and single deletion mutant). The observed response differs from *A. tumefaciens and R. legumniosarum*, where only the external addition of AI caused an increased copy number or *repA* gene transcription, respectively (Pappas and Winans, 2003a; McAnulla et al., 2007; Pappas, 2008). Thus, the observation made here for NGR234 was unexpected and indicated that possibly different or additional regulatory elements were involved in copy number control of pNGR234*a* compared to the well-characterized *A. tumefaciens* or *R. leguminosarum* systems.

### Two new, previously not detected ORFs within the trai-repA intergenic region

**Figure 2:**
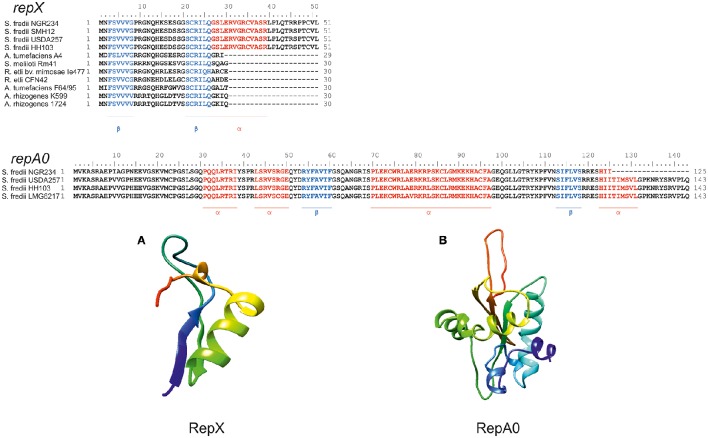
**Amino acid ClustalW Alignments of ***RepX*** (A)** and *RepA0* **(B)** and structure models of both proteins. Both models predict folded proteins with ß-sheets, α-helical and coiled regions. In the alignments (upper panel), red amino acids display probable components of α-helices and blue amino acids display components of β-sheets. In the 3D structures depicted in the lower panel the RepA0 3D model predicts two antiparallel-ß-sheets and two α-helical areas. The RepX model predicts one ß-sheet and one α-helical area. The analysis and sequence alignments were done with PROMALS3D (http://prodata.swmed.edu/promals3d/promals3d.php) and the 3D structure prediction was done using the web tool available at http://robetta.bakerlab.org.

Because of the above described increase in copy number of pNGR234*a* in the double deletion mutant and in the single *traI* mutant we asked, whether in NGR234 the *repA* or flanking genes, such as *traI* would be different from those of other known *repABC* plasmids (Figure 1A). A more detailed analysis of the pNGR234*a traI-repA* intergenic region indicated that the region is twice as large as observed for *A. tumefaciens* and most other rhizobial isolates carrying *repABC*-type plasmids (Figure 1A, Table 3). We observed that in NGR234 and the two other broad host range strains, HH103 and USDA257, an 803-bp intergenic region separates *traI* and *repA*. In *A. tumefaciens, S. meliloti*, and *R. etli* this intergenic region is usually not larger than 376 bp and only in *R. rhizogenes* NBRC13257 a 441-bp intergenic region was observed (Table 3). In NGR234, USDA257 and HH103 this intergenic region codes for two putative open reading frames. We have designated these genes *repX* and *repA0* (Figure 1A). *RepX* probably codes for a 51 aa protein and *repA0* for a 143 aa protein. The analysis of both sequences with PROMALS3D (http://prodata.swmed.edu/promals3d/promals3d.php) revealed for the longer variant of *repX two* β-sheet-regions and one α-helix-region and in case of *repA0* two β-sheets and four α-helix secondary structure regions (Figure 2). This observation was supported by data using 3D structure prediction tools.

No known conserved domains could be detected in either peptide sequence with the conserved domain search provided by NCBI (http://www.ncbi.nlm.nih.gov/Structure/cdd/wrpsb.cgi). Although neither gene has been reported in other rhizobial species a detailed analysis of the intergenic region of *A. tumefaciens* nor others indicated that *repX* is present in some of the closely related species but often in a truncated version. The *repA0* gene could not be identified in other strains (Figure 2). Notably, the expression of *repA0* on a self-replicable plasmid resulted in a 3.1–3.8-fold increased copy number of pNGR234*a* (Table 2). However, overexpression of *repX* on a self-replicable plasmid had no influence on the pNGR234*a* copy number (Table 2). Similarly, no influence of the empty vector on the pNGR234*a* copy number was observed. We further asked if *repA0* is transcribed separate from *repA* as an independent gene. Therefore, another RT-PCR assay on cDNA level was performed using primers indicated in table S2. This test indicated that *repA0* is transcribed as distinct gene and not as part of *repA* (Figure 1C).

### RNA-seq data imply a role of repA0 in copy number control of pNGR234a

To further estimate the effects of the complete absence of AI on the metabolism of NGR234, we used RNA-seq in the background of the NGR234-Δ*traI*-Δ*ngrI* double deletion mutant and compared the data to the parent strain and to our previous data sets obtained for the NGR234-Δ*traI* and the NGR234-Δ*ngrI* single deletion mutants (Krysciak et al., 2014).

In the analysis of RNA-seq data we considered genes with a fold-change of ≥ 2.0 and an adjusted *p*-value of ≤ 0.05 as statistically significant and differentially expressed between two distinct setups. Only those values that complied with both requirements were used for subsequent analyses. The differentially regulated genes are given in supplementary table 4.

Altogether more than 1261 genes were differentially regulated in the double deletion mutant compared to the wild type strain. This equals almost 20% of all predicted genes. Of these, the majority was upregulated (75%) and regulated through sense transcripts (68% of all regulated genes). Given the large differences in size of the three replicons, we observed a rather unequal distribution of the regulated genes over the three replicons (pNGR243*a*–32.43%; pNGR243*b*–21.33%; cNGR234–46.24%). Additionally, our data indicated that of the 418 previously predicted genes on pNGR234*a* 408 (98%) were upregulated. For 357 of the 408 genes the sense and anti-sense transcripts were increased (Supplementary Table 4). In contrast, on pNGR234*b* 269 genes (11.5%) were regulated and only two of these genes were simultaneously regulated via sense and antisense transcripts. Similarly, on the chromosome 583 genes (16.05%) were regulated and 15 genes were simultaneously regulated via sense and antisense transcripts. Part of the transcriptome results were verified via qPCR (Supplementary Tables 5, 6). Within this framework, the most surprising observation was that virtually all the pNGR234*a*-encoded genes were transcribed at significantly higher levels. The average of the pNGR234*a* genes was increased by a factor of 7. The data obtained using RNA-seq indicated that in the double deletion mutant NGR234-Δ*traI*-Δ*ngrI* many more genes are differentially regulated than in any of the previously reported single AI synthase deletion mutants compared to the parent strain. However, the *repABC* genes showed a 23-43-fold increased level of transcription. Notably, the *repA0* and *repX* genes showed a 107.11 and 656.39-fold increased transcription, respectively (Figure 1B, Table 4 and Supplementary Table 4). It is further noteworthy that the transcription of the two predicted ncRNAs (NGR_a00030, an antisense RNA regulator of the RepB translation and NGR_a00040, a negative antisense RNA regulator of RepC) flanking *repB* and *repC* were also strongly upregulated in the double deletion mutant. NGR_a00030 is most likely a homolog of *repE/inc*α. Thus, we conclude the high level transcription of *repABC* is probably responsible for the increased copy number of pNGR234*a* in the double deletion strain or vice versa. It is notable, that the strong increase in *repX, repA0* and *repA-C* transcription was not observed in the single deletion mutants of *traI* or *ngrI* as previously published (Krysciak et al., 2014).

**Table 4:** **Fold-increase of genes associated with the ***repABC*** locus replication in the background of the NGR234-Δ***traI***-Δ***ngrI*** mutant compared to the wild type strain and as estimated using RNA-seq**.

**Start**	**End**	**Strand**	**Orientation**	**Locus_tag**	**Gene**	**Protein function**	**Fold Change**
534836	535462	-	anti-sense	NGR_a04220	*traI*	Autoinducer synthase TraI	16.74
535461	535617	+	sense	NGR_a04230	*repX*	hypothetical	656.39
535839	104	+	sense	NGR_a04240	*repA0*	hypothetical, pNGR234*a* copy number control	107.11
101	1324	+	sense	NGR_a00010	*repA*	replication protein RepA	23.97
101	1324	+	anti-sense	NGR_a00010	*repA*	replication protein RepA	2.07
1381	2361	+	sense	NGR_a00020	*repB*	replication protein RepB	33.57
1381	2361	+	anti-sense	NGR_a00020	*repB*	replication protein RepB	2.90
2363	2409	-	anti-sense	NGR_a00030	ncRNA	antisense regulator of RepB	29.47
2363	2409	-	sense	NGR_a00030	ncRNA	antisense regulator of RepB	18.19
2450	2518	-	anti-sense	NGR_a00040	ncRNA	negative antisense regulator of RepC	42.37
2516	3730	+	sense	NGR_a00050	*repC*	replication initiation protein RepC	43.85
2516	3730	+	anti-sense	NGR_a00050	*repC*	replication initiation protein RepC	3.15

*Sequence positions refer to Genbank entries U00090 and BK009410*.

### Reporter gene studies, qRT-PCR and immunoblotting confirm the elevated transcription of nod, nop, repA, and NGR_a00860 gene expression

Further tests with reporter strains using the *Escherichia coli lacZ* gene fused to the *nodABC* promoter and the *nopB* promoter confirmed the elevated expression of these genes (Table 5). The expression was most pronounced after 120 h of growth. However, after 24 and 48 h of growth already significant (2-fold) differences between the parent strain and the double deletion mutant were observed with respect to the *nodABC* gene expression. For the *nopB* reporter the differences were only visible after 48 h (>2-fold). After 72 h, we observed an eight-fold increased transcription in the double deletion mutant in the absence of apigenin vs. the wild type strain (Table 5). In the presence of apigenin, the transcription in the mutant was 3.7-fold higher compared to the induced wild type. Similar expression levels were observed for the *nopB* reporter fusion where we observed a 20-fold higher level of *lacZ* expression in NGR234-Δ*traI*-Δ*ngrI* compared to the untreated parent strain; and in the presence of apigenin the mutant produced 333-fold more ß-galactosidase activity compared to the wild type (Table 5). Altogether these data implied that in the absence of any AI an increased level of transcription of the pNGR234*a* genes can be observed. Further qRT-PCR test results confirmed largely the above described findings (Supplementary Tables 5, 6).

**Table 5:** **β-galactosidase activities of NGR234 and NGR234-ΔtraI-Δ***ngrI*** carrying either a ***nodABC::lacZ*** or a ***nopB::lacZ*** promoter fusion and in response to apigenin**.

**Rhizobium (construct)**	**Time Period (hours)**				
	**24**	**48**	**72**	**96**	**120**
NGR234 (p*nodABC*)	79.32±9.9	55.45±2.41	11.75±1.87	13.08±0.59	5.97±0.64
NGR234 (p*nodABC*) + apigenin	1597.05±170.32	1518.06±101.32	907.16±212.13	785.80±25.78	528.24±232.87
NGR234 (p*nopB*)	146.12±0.77	54.98±8.49	11.46±0.75	9.87±0.04	2.50±0.20
NGR234 (p*nodB*) + apigenin	1238.19±60.35	1086.54±51.51	86.98±55.71	84.75±2.21	5.23±0.0
*ngr*IΔ/*tra*IΔ (pnodABC)	147.07±2.00	92.18±2.02	53.21±05.00	71.02±0.67	47.35±0.45
*ngr*IΔ/*tra*IΔ (pnodABC) + apigenin	2430.34±253.73	3090.85±35.53	2299.20±1.74	2026.67±16.51	1952.91±0.41
*ngr*IΔ/*tra*IΔ (pnopB	125.97±16.48	119.69±0.41	65.22±5.52	62.2±3.06	52.12±0.91
*ngr*IΔ/*tra*IΔ (pnodB) + apigenin	670.58±199.42	2684.47±6.83	2023.53±29.54	1835.47±8.95	1733.38±19.18

*Data are mean values with the corresponding standard deviation of 3 measurements. Promoters fused to the ß-galactiosdase gene are given in brackets as pnodABC or pnopB*.

**Figure 3:**
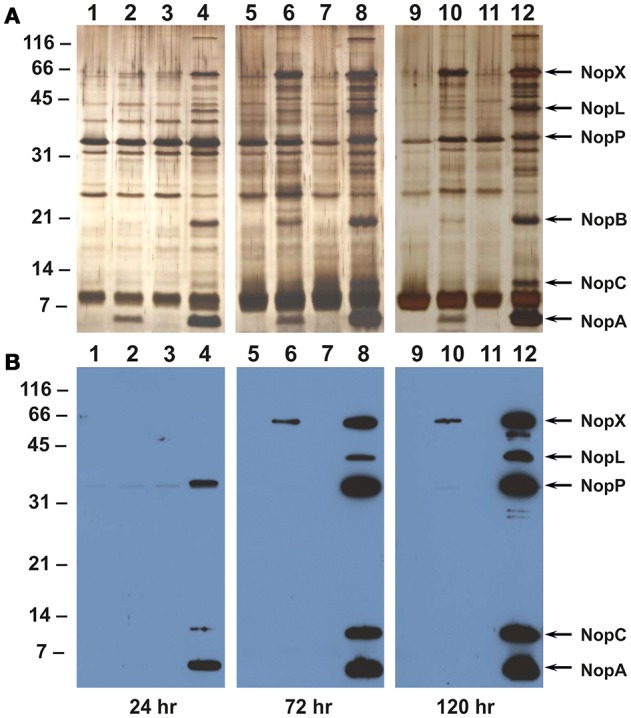
**Extracellular protein profile and immunoblot analysis of ***Sinorhizobium fredii*** NGR234 and NGR234-Δ***traI***-Δ***ngrI*** double deletion mutant**. Extracellular proteins isolated from cells grown in the absence (odd number lanes) or presence (even numbered lanes) of 1 μM apigenin were resolved by 15% SDS-PAGE and silver stained **(A)** or transferred to nitrocellulose membrane **(B)** for immunological analysis with a cocktail of antibodies raised against the individual Nop proteins. Lanes 1–4 contain protein samples harvested from 24 h, lanes 5–8 from 72 h, and lanes 9–12 from120 h old cultures. The size of the molecular weight markers in kDa and the identity of the immunoreactive proteins are also shown.

Additionally, we analyzed the extracellular protein profile of the NGR234 wild type and NGR234-Δ*traI*-Δ*ngrI* with respect to the production of selected Nop proteins. Nop proteins are secreted through the type 3 secretion system (T3SS-I), which is encoded on pNGR234*a*. Their expression is under control of a Nod factor-dependent promoter (Deakin and Broughton, 2009). Nop proteins in the supernatants were detected using a cocktail of highly specific antibodies as previously described (Krishnan et al., 2003). These immunoblot analyses further indicated that Nops are produced and secreted in the mutant strain at higher levels compared to the parent strain (Figure 3). Consistent with the ß-galactosidase data the highest expression of Nops was detected in the mutant strain after 120 h and in the presence of apigenin. No Nops were detected in the cultures that were not treated with apigenin. Altogether these tests support the above described findings with respect to the increased transcription of the pNGR234*a*-harbored genes in NGR234-Δ*traI*-Δ*ngrI*.

### NGR234-ΔtraI-ΔngrI induces root hair curling in the absence of apigenin

**Figure 4:**
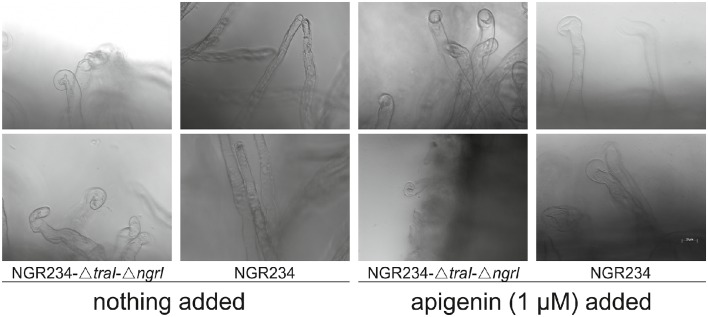
**Root hair curling assays using Nod factor extracts of ***Sinorhizobium fredii*** NGR234 and the NGR234-Δ***traI***-Δ***ngrI*** double deletion mutant**. Images in the upper and lower panel show root hair curling after 24 h induction with extracts of cultures treated either with 1 μM apigenin or the non-treated controls for the parent strain and the NGR234-Δ*traI*-Δ*ngrI* double deletion mutant.

An early response to plant released flavonoids is the activation of the *nod* gene transcription, which leads to the production of Nod factors. The release of these factors induces, through a Nod factor specific signal cascade, the curling of root hairs, which initiates the symbiosis process (D'Haeze and Holsters, 2002). Up to now it was thought that the symbiosis in the *Alphaproteobacteria* can only be initiated by the activation of *nod* gene transcription induced through flavonoids or related plant-released molecules and salt stress (Mulligan and Long, 1985; Rossen et al., 1985). In the light of the observations described above, we asked if the elevated transcription of all genes necessary for the first symbiosis steps on pNGR234*a* in NGR234-Δ*traI*-Δ*ngrI* would be sufficient to allow production of Nod factors. Therefore, we extracted Nod factors from culture supernatants of the wild type strain and the double deletion mutant grown with and without apigenin, which was chosen as a positive control that is known to induce the transcription of symbiosis-related genes. These tests indicated that Nod factors produced from non-induced NGR234-Δ*traI*-Δ*ngrI* cells were sufficient to induce root hair curling on germinating *Vigna unguiculata* roots. In contrast, when we tested Nod factor extracts of non-induced wild type cells, virtually no root hair curling was observed after a 24 h co-incubation time period. A further test implied that mutant cells treated with 1 μM apigenin and the wild type as positive controls induced root hair curling after 24 h (Figure 4).

Altogether these data imply that in NGR234 the absence of HSL molecules causes an increased copy number of pNGR234*a* and an elevated transcription of the symbiotic genes and this allows sufficient Nod factor production to induce root hair curling even in the absence of a plant-released flavonoid, such as apigenin. In our view these data again provide strong evidence for the elevated transcription of symbiotic genes in NGR234 in the absence of the AI molecules produced by TraI and NgrI.

## Discussion

Flavonoids, isoflavonoids, betaines and high concentrations of NaCl induce the transcription of nodulation (*nod, noe, and nol*) genes (Djordjevic et al., 1986; Firmin et al., 1986; Peters et al., 1986; Kosslak et al., 1987; Phillips et al., 1992; Guasch-Vidal et al., 2013). Thereby, the plant-released polyphenols and the betaines are highly specific signals that are perceived by the bacterial NodD or SyrM receptor and activator proteins, which in turn activate transcription of many symbiosis related genes (Hartwig et al., 1990; Swanson et al., 1993; Barnett and Long, 2015). The *nod* and *nol* genes encode for the strain-specific biosynthesis of chito-lipo-oligosaccharides, commonly called Nod factors. The bacterially synthesized Nod factors are released and induce root hair curling on susceptible legume plants. They are also assumed to be highly specific signals that are key determinants for the host range (Broughton and Perret, 1999; Geurts and Bisseling, 2002; Deakin and Broughton, 2009).

Within this manuscript we have reported on a flavonoid—and betaine-independent transcription of all symbiotic genes located on pNGR234*a* of the broad host range strain NGR234. This phenomenon was mainly observed in the double AI-synthase deletion mutant NGR234-Δ*traI*-Δ*ngrI*. This mutant strain is not capable to produce any HSL-like AI molecules (Krysciak et al., 2014). Since the copy number of pNGR234*a* was increased in the double deletion mutant to 3–8-copies per cell (Table 1), it implies that AI molecules attenuate gene expression of pNGR234*a*-born genes by strictly maintaining one copy per cell. Interestingly, Mc Anulla et al. reported earlier an increase in *repA* transcription in *R. leguminosarum* in response to the presence of the native AI molecule (McAnulla et al., 2007). Thus, their observation fits well with our results obtained in response to the addition of extra copies of AI synthase genes (Table 2). However, the observation that the complete absence of HSL-like AI molecules increases the copy number of pNGR234*a* is novel.

The increased transcription of the genes located on pNGR234*a* in the double deletion mutant was linked to two new ORFs designated *repX* and *repA0* that were located in the *traI*-*repA* intergenic region. Both ORFs probably encode for small proteins (*repX* for 51 and *repA0* for 143 amino acids) and have previously not been described. Overexpression of *repA0* increased the copy number of pNGR234*a* in the NGR234 wild type to 3–4 copies per cell, suggesting a regulatory role for this ORF. Within this framework, it is notable that not much is known on the regulation of the copy number in NGR234. However, in the closely related *A. tumefaciens* the TraR- and QS-dependent expression of the *repABC* genes has been studied extensively (Pappas and Winans, 2003a; Cho and Winans, 2005; Pinto et al., 2012). In *A. tumefaciens* strains the *repABC* genes together with a small non-coding RNA (ncRNA; *repE*/incα) are responsible for the copy number control of the corresponding *repABC*-type and tumor inducing Ti plasmid. Thereby, increased levels of AI or plant-released nopaline induce an elevated transcription of the *repABC* genes resulting in a 5-8-fold increased copy number. Furthermore, the increased copy number corresponds with a 3-4-fold increase in tumorigenesis (Li and Farrand, 2000; Pappas and Winans, 2003b; Pappas, 2008). In addition to the QS-dependent copy number control, phenolic compounds released by plants have influence on the copy number via the two component regulatory system VirA/VirG (Pappas and Winans, 2003a; Cho and Winans, 2005; Pinto et al., 2012; Subramoni et al., 2014).

**Figure 5:**
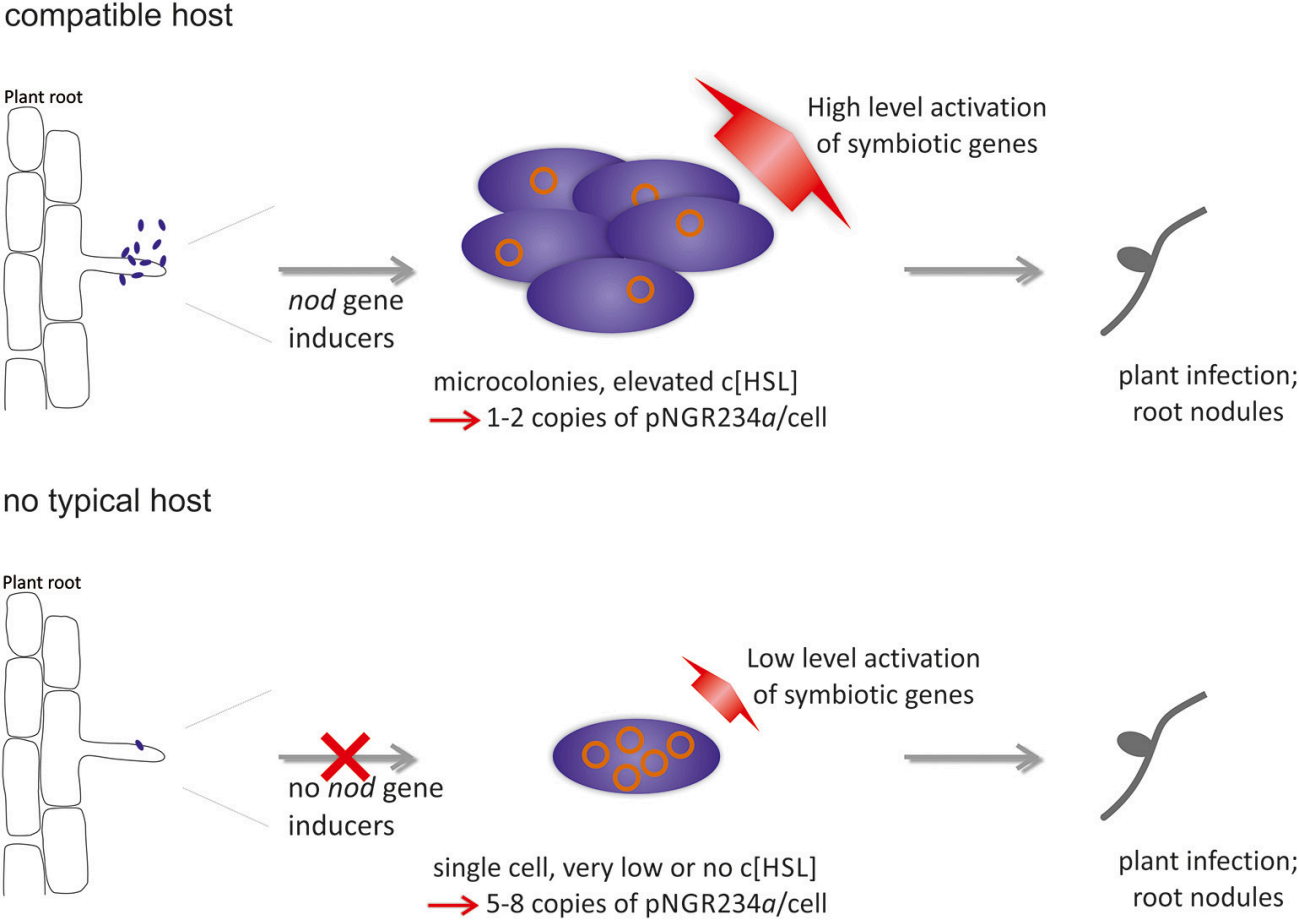
**Possible model of alternative ***nod*** gene induction in ***Sinorhizobium fredii*** NGR234 and related broad host range rhizobia**. Image in the upper panel depicts the classical *nod* gene-inducing pathway, during which plant phenolic compounds and betaines are required as signals for induction of genes with relevance to plant infection and symbiosis. Once the signal is perceived Nod factors are produced and root nodule formation can be initiated. The lower panel describes a simpler pathway that is employed if no compatible host is found and if only single cells are attached to the root hairs. Under these conditions, the absence of autoinducer molecules triggers an increase in copy number of the symbiotic replicon from 1 copy to 5–8 copies per cell. This in turn results in a low level transcription of all genes located on the symbiotic plasmid and allows the production Nod factors and T3SS effectors.

We observed a transcription of virtually all pNGR234*a*-born genes in the absence of plant-released compounds to be high enough to allow Nod factor production and induce root hair curling in NGR234-Δ*traI*-Δ*ngrI* (Tables 4, 5 and Figure 4). In addition, Nops (nodulation outer proteins) were produced in a higher concentration in the induced double deletion mutant compared to the induced wild type strain (Figure 3). Altogether these findings suggest that NGR234 has evolved an alternative mechanism that allows the microbe to initiate the nodulation process irrespective of the presence of specific flavonoids or other *nod*-gene inducing plant compounds. It further implies that the absence of AI molecules triggers this response. From an ecological point of view it is likely that mainly single cells infect the root hair rather than high-density populations producing high levels of AI molecules. This can be deduced from the observation that overexpression of quorum quenching genes in the rhizospheres has no symbiotic phenotype and transcripts of the AI synthases are not changed in root nodules (Krysciak et al., 2011; Li et al., 2013). Thus, under rhizosphere conditions it might be advantageous for NGR234 to recognize the lack of AI molecules as an activation signal for increasing the copy number of its symbiotic replicon. The increase in copy number turns on all symbiotic genes in a low level and, as a consequence, the microorganism can infect plant roots even though the matching phenolic or betaine signal molecule is absent. This possible scenario is outlined in Figure 5. The concept of increased copy numbers in the absence of AI molecules makes sense in two ways. First, it helps to overcome a shortage in AI molecules due to the single cell status and second, it allows transcription of the essential and infection-related genes in the absence of a host-specific signal. Under these conditions even a non-host plant will probably be infected. Of course, whether the initial infection results in effective nodules afterwards depends on many plant-associated factors.

Furthermore, it is reasonable to hypothesize that the observation made here could be a general strategy that is employed by other pathogenic or symbiotic bacteria carrying self-replicable plasmids harboring the respective infection-related genes. In fact, very recently a similar phenomenon was published for the human pathogen *Yersinia pseudotuberculosis*. Similar to NGR234, this bacterium carries a type 3 secretion system (T3SS), which is essential for infection, on a 70 kb virulence IncFII-class plasmid. While in *Y. pseudotuberculosis* the T3SS is usually expressed during host cell contact, this can be overcome by incubation at 37°C and Ca^2+^-deficiency. Interestingly, in *Y. pseudotuberculosis* 37°C and Ca^2+^-deficiency induce up-regulation of the virulence plasmid copy number and this causes expression of the T3SS. Thereby, the two small proteins CopA and YopD appear to play an essential role in copy number regulation (Wang et al., 2016). The findings reported for NGR234 and for *Y. pseudotuberculosis* imply that host-associated bacteria have evolved alternative ways to ensure successful infection of the host even in the absence of the corresponding host-specific signals by increasing the copy number of the respective replicon. Therefore, the observation made in this manuscript that NGR234 induces root hair curling in the absence of plant-compounds and in the absence of AI molecules suggests that this may indeed be another key to broad host range.

Finally, the observations made here will give new clues to engineering bacterial strains that can infect and establish a nitrogen-fixing symbiosis with non-legumes in the absence of plant flavonoids and other essential inducers. These findings could be relevant for ensuring the global nitrogen supply of important non-legume crops.

## Author contributions

KF and DK performed the RNA-seq experiments and their analyses; HK did the promoter studies, Nop analyses and wrote and edited the manuscript; JG, DK, and WS designed, wrote and edited the manuscript. CS also contributed to the study. JG performed the qPCR experiments, established deletion mutants and the root hair curling assay. KP helped with the root hair curling assays. SG carried out parts of the graphical analysis of the RNA-seq data. HK and NK provided 3-D models for the amino acid sequences and further analyses of the small peptides. HS and NK contributed to structural analysis.

## Funding

This work was kindly funded by the Deutsche Forschungsgemeinschaft through the grant STR451/7-1 within the SPP1617 priority program.

## Conflict of interest statement

The authors declare that the research was conducted in the absence of any commercial or financial relationships that could be construed as a potential conflict of interest.
