# Supplementary material for: The Absence of the N-acyl-homoserine-lactone Autoinducer Synthase Genes traI and ngrI Increases the Copy Number of the Symbiotic Plasmid in Sinorhizobium fredii NGR234
Source: Front Microbiol. 2016 Nov 18;7:1858. doi: 10.3389/fmicb.2016.01858 (PMC5114275; doi:10.3389/fmicb.2016.01858)
Supplement: Supplementary file 1 [file Table1.docx]

**Supplementary Table S1.** Primers used in this study.

| **Oligonucelotide** | **Sequence 5’-3’^a^** | **Size (bp)** | **Target region/description** |
| --- | --- | --- | --- |
| repA_int_fw | CAACCGCAGCAACTCAGAAC | 20 | amplification of parts of *repA* |
| repA_int_rev | ATTCTCGCCGACTGACAAGG | 20 |  |
| RT_a00010_fw | GCAGCAGTTCCCACCGAATG | 20 | amplification of parts of *repA* and qRT-PCR for RNA-seq verification |
| RT_a00010_rev | GCACGTAGTTCCTGGCTTCC | 20 |  |
| repX_int_fw | ATGAATTTCTCCGTCGTTGTTG | 22 | amplification of *repX* |
| repX_int_rev | CTACAAGACGCAAGGGGGCCTTC | 23 |  |
| RT_recA_fw | CGGCTCGTAGAGGACAAATCG | 21 | reference gene 1 qRT-PCR |
| RT_recA_rev | CAATGATGCGCCCTTTCGG | 19 |  |
| RT_rpoD_fw | ACATCACCAATGTCGGCGGTGAAG | 24 | reference gene 2 qRT-PCR |
| RT_rpoD_rev | TGCAGCTTGCGGAGCTTCTTGTAG | 24 |  |
| RT_c24920_fw | GCCCGCACAAGAGGCAATTCTAGA | 24 | qRT-PCR for RNA-seq verification |
| RT_c24920_rev | GCGATCCAGGAACATGGCGTTCA | 23 |  |
| RT_a00860_fw | CCCGGTTTACTCGAGCTGTC | 20 | qRT-PCR for RNA-seq verification |
| RT_a00860_rev | GCTGCTCCTCCGTAACTGTG | 20 |  |
| RT_c09750_fw | CAATTCGAAAGTGATCTTCGGGCAGCA | 27 | qRT-PCR for RNA-seq verification |
| RT_c09750_rev | GAGCCACATCGACTCGGCGTAT | 22 |  |
| RT_a00550_fw | AGGTACTCTCACGAGTGCAGTTGG | 24 | qRT-PCR for RNA-seq verification |
| RT_a00550_rev | CTGTACGCGTTCATCCGCCG | 20 |  |
| RT_a00440_fw | ACCGCCTTGCTGCACTTCAGCAAT | 24 | qRT-PCR for RNA-seq verification |
| RT_a00440_rev | CGTCCATTTCCATGCCGTCGACAA | 24 |  |
| RT_c17900_fw | ATGAACACGCTCGCCTATCTCATCC | 25 | qRT-PCR for RNA-seq verification |
| RT_c17900_rev | TCAATGCCTGTCCGGCTTGCCAT | 23 |  |
| RT_c09120_fw | ACCAAACTGTCGCTCGCGCACAA | 23 | qRT-PCR for RNA-seq verification |
| RT_c09120_rev | CAGGTAAAGGGCGATGTCGAAGCT | 24 |  |
| TraR_up_fw | aaatctagaCTCTCCTATCTTCTTGACCG | 29 | amplification of *traR* upstream flank |
| TraR_up_rev | aaaggatccTGTGGCGCTACAATCACTG | 28 |  |
| TraR_do_fw | aaaggatccTGTAGCGGCTAGACCGATACAATG | 33 | amplification of *traR* downstream flank |
| TraR_do_rev | aaatctagaGTTCTAGTTGTCATCGTCGAAAGGC | 34 |  |
| TraM_up_fw | aaaggatccCGCTCGATTTTACCCTGCAGTAG | 32 | amplification of *traM* upstream flank |
| TraM_up_rev | aaatctagaCCGCTTCTCCCTGATTTGATTTCC | 33 |  |
| TraM_do_fw | aaatctagaGACAACTAGAACTCAGACCAGG | 31 | amplification of *traM* downstream flank |
| TraM_do_rev | aaaggatccGCGGTCAAGAAGATAGGAG | 28 |  |

1. Restriction sites are underlined
