# Supplementary material for: The Absence of the N-acyl-homoserine-lactone Autoinducer Synthase Genes traI and ngrI Increases the Copy Number of the Symbiotic Plasmid in Sinorhizobium fredii NGR234
Source: Front Microbiol. 2016 Nov 18;7:1858. doi: 10.3389/fmicb.2016.01858 (PMC5114275; doi:10.3389/fmicb.2016.01858)
Supplement: Supplementary file 2 [file Table2.docx]

**Supplementary Table S2.** Overall transcriptome statistics for the analyzed NGR234 and NGR234-Δ*traI*-Δ*ngrI*.

| **Sample**  **#** | **NGR234**  **genotype** | **OD**_600_ **after 48 h growth** | **Treatment^a^** | **No. of reads generated**  X10^6^ | **No. of uniquely mapped reads**  X10^6^ |
| --- | --- | --- | --- | --- | --- |
| 1 | wt | 9.10 | none | 1.36 | 0.55 |
| 2 | wt | 8.96 | none | 2.48 | 1.57 |
| 3 | wt | 8.46 | none | 2.95 | 1.75 |
| 4 | wt | 7.76 | apigenine (1 µM) | 3.45 | 2.21 |
| 5 | wt | 8.10 | apigenine (1 µM) | 2.36 | 1.47 |
| 6 | wt | 6.84 | apigenine (1 µM) | 2.35 | 1.42 |
| 7 | Δ*traI*-Δ*ngrI* | 4.12 | none | 3.57 | 1.72 |
| 8 | Δ*traI*-Δ*ngrI* | 4.69 | none | 3.44 | 1.92 |
| 9 | Δ*traI*-Δ*ngrI* | 4.21 | none | 2.66 | 1.39 |
| 10 | Δ*traI*-Δ*ngrI* | 3.47 | apigenine (1 µM) | 2.96 | 1.20 |
| 11 | Δ*traI*-Δ*ngrI* | 3.24 | apigenine (1 µM) | 3.39 | 1.62 |
| 12 | Δ*traI*-Δ*ngrI* | 3.12 | apigenine (1 µM) | 3.31 | 1.72 |
| 13 | wt | 5.92 | none | 2.35 | 1.32 |
| 14 | wt | 7.92 | none | 3.77 | 2.15 |
| 15 | wt | 6.72 | none | 2.55 | 1.56 |

^a^ Controls for apigenin samples were supplemented with an equal amount of KOH
