# Supplementary material for: The Absence of the N-acyl-homoserine-lactone Autoinducer Synthase Genes traI and ngrI Increases the Copy Number of the Symbiotic Plasmid in Sinorhizobium fredii NGR234
Source: Front Microbiol. 2016 Nov 18;7:1858. doi: 10.3389/fmicb.2016.01858 (PMC5114275; doi:10.3389/fmicb.2016.01858)
Supplement: Supplementary file 4 [file Table4.docx]

**Supplementary Table S4: qRT-PCR verification of RNA-seq data obtained for the NGR234-△traI-△ngrI mutant vs. the NGR234 wildtype strain.** Green colored numbers and arrow indicate an upregulation; red number and downwards oriented arrows indicate downregulation. Data are mean values of 3 experiments and standard deviations are given in brackets.

| **Target gene** | **RNA-seq results** | **qPCR results** |
| --- | --- | --- |
| NGR_c24920 | 9.0 ↓ | 1.9 (± 0.07) ↓ |
| NGR_a00860 | 24.4 ↑ | 1.1 (± 0.19) ↑ |
| NGR_c09750 | 7.1 ↓ | 1.1 (± 0.14) ↓ |
| NGR_a00010 | 24 ↑ | 3.9 (± 0.45) ↑ |
