# Supplementary material for: The Absence of the N-acyl-homoserine-lactone Autoinducer Synthase Genes traI and ngrI Increases the Copy Number of the Symbiotic Plasmid in Sinorhizobium fredii NGR234
Source: Front Microbiol. 2016 Nov 18;7:1858. doi: 10.3389/fmicb.2016.01858 (PMC5114275; doi:10.3389/fmicb.2016.01858)
Supplement: Supplementary file 5 [file Table5.docx]

**Supplementary Table S5: qRT-PCR verification of RNA-seq data obtained for NGR234-△*traI*-△*ngrI* treated with 50 µM apigenin compared to untreated NGR234-△*traI*-△*ngrI* cells.** Green colored numbers and arrow indicate an upregulation; red number and downwards oriented arrows indicate downregulation. Data are mean values of 3 experiments and standard deviations are given in brackets.

| **Target gene** | **RNA-seq results** | **qPCR results** |
| --- | --- | --- |
| NGR_a00550 | 28.0 ↑ | 68.9 (± 11.63) ↑ |
| NGR_a00440 | 35.3 ↑ | 8.1 (± 1.32) ↑ |
| NGR_c17900 | 14.0 ↓ | 11.4 (± 0.07) ↓ |
| NGR_c09120 | 21.6 ↑ | 39.6 (± 8.66) ↑ |
