# Supplementary material for: The Absence of the N-acyl-homoserine-lactone Autoinducer Synthase Genes traI and ngrI Increases the Copy Number of the Symbiotic Plasmid in Sinorhizobium fredii NGR234
Source: Front Microbiol. 2016 Nov 18;7:1858. doi: 10.3389/fmicb.2016.01858 (PMC5114275; doi:10.3389/fmicb.2016.01858)
Supplement: Supplementary file 6 [file Table6.docx]

**Supplementary Table S6: Bacterial strains and plasmids used in this study.**

| **Bacterial strain/plasmid** | **Description** | **abbreviation in the text** | **source/reference** |
| --- | --- | --- | --- |
| Bacterial strains |  |  |  |
| *Sinorhizobium fredii*  NGR234 | wild type strain | NGR234 | (Trinick 1980) |
| *Sinorhizobium fredii* NGR234-△*traI*-△*ngrI* | *Sinorhizobium fredii* NGR234 deleted in both *luxI-*like autoinducer synthase genes *traI* and *ngrI* | NGR234-△*traI*-△*ngrI* | (Krysciak *et al.* 2014) |
| *Sinorhizobium fredii* NGR234-△*traI*-△*ngrI*_c*traI*c*ngrI* | *Sinorhizobium fredii* NGR234 deleted in both autoinducer synthase genes *traI* and *ngrI* and carrying the complementation plasmid pBBR1MCS-2::c*traI*c*ngrI* | NGR234-Δ*traI*-Δ*ngrI*_c*traI*c*ngrI* | This study |
| *Sinorhizobium fredii* NGR234-△*traI* | *Sinorhizobium fredii* NGR234 deleted in the autoinducer synthase gene *traI* | NGR234-△*traI* | (Krysciak *et al.* 2014) |
| *Sinorhizobium fredii* NGR234-△*traI*_c*traI* | *Sinorhizobium fredii* NGR234 deleted in the autoinducer synthase gene *traI* and carrying the complementation plasmid pBBR1MCS-2::c*traI* | NGR234-△*traI*_c*traI* | (Krysciak *et al.* 2014) |
| *Sinorhizobium fredii* NGR234-△*ngrI* | *Sinorhizobium fredii* NGR234 deleted in the autoinducer synthase gene *ngrI* | NGR234-△*ngrI* | (Krysciak *et al.* 2014) |
| *Sinorhizobium fredii* NGR234-△*ngrI*_c*ngrI* | *Sinorhizobium fredii* NGR234 deleted in the autoinducer synthase gene *traI* and carrying the complementation plasmid pBBR1MCS-2::c*ngrI* | NGR234-△*ngrI*_c*ngrI* | (Krysciak *et al.* 2014) |
| *Sinorhizobium fredii* NGR234-△*traM* | *Sinorhizobium fredii* NGR234 deleted in the antiactivor gene *traM* | NGR234-△*traM* | This study |
| *Sinorhizobium fredii* NGR234-△*traR* | *Sinorhizobium fredii* NGR234 deleted in the regulator gene *traR* | NGR234-△*traR* | This study |
| Plasmids |  |  |  |
| pBBR1MCS-2 | broad host range vector |  | (Kovach *et al.* 1995) |
| pBBR1MCS-2::c*traI*c*ngrI* | plasmid harboring the genes for both autoinducer synthases *traI* and *ngrI* |  | This study |
| pBBR1MCS-2::c*traI* | plasmid harboring the gene for the autoinducer synthase *traI* |  | (Krysciak *et al.* 2014) |
| pBBR1MCS-2::c*traI* | plasmid harboring the gene for the autoinducer synthase *ngrI* |  | (Krysciak *et al.* 2014) |
| pBBR1MCS-2::*repX* | plasmid harboring the *repX* gene |  | This study |
| pBBR1MCS-2::*repA0* | plasmid harboring the *repA0* gene |  | This study |
| pNPTS138-R6KT | suicide plasmid for the construction of deletion mutants |  | (Lassak *et al.* 2010) |
| pNPTS138-R6KT::△*traR* | suicide plasmid harboring the ligated flanks of the *traR* gene for the deletion of the *traR* gene in NGR234 |  | This study |
| pNPTS138-R6KT::△*traM* | suicide plasmid harboring the ligated flanks of the *traM* gene for the deletion of the *traM* gene in NGR234 |  | This study |

References

**Kovach ME, Elzer PH, Hill DS, Robertson GT, Farris MA, Roop RM, 2ndandPeterson KM**. 1995. Four new derivatives of the broad-host-range cloning vector pBBR1MCS, carrying different antibiotic-resistance cassettes. Gene **166**(1): 175-176.

**Krysciak D, Grote J, Orbegoso MR, Utpatel C, Forstner KU, Li L, Schmeisser C, Krishnan HBandStreit WR**. 2014. RNA sequencing analysis of the broad-host-range strain *Sinorhizobium fredii* NGR234 identifies a large set of genes linked to quorum sensing-dependent regulation in the background of a *traI* and *ngrI* deletion mutant. Applied and Environmental Microbiology **80**(18): 5655-5671.

**Lassak J, Henche AL, Binnenkade LandThormann KM**. 2010. ArcS, the cognate sensor kinase in an atypical Arc system of *Shewanella oneidensis* MR-1. Applied and Environmental Microbiology **76**(10): 3263-3274.

**Trinick MJ**. 1980. Relationships amongst the fast-growing *Rhizobia* of *Lablab purpureus*, *Leucaena leucocephala*, *Mimosa* spp., *Acacia farnesiana* and *Sesbania grandiflora* and their affinities with other rhizobial groups. Journal of Applied Microbiology **49**(1): 39-53.
