# Supplementary figures and images for: The Absence of the N-acyl-homoserine-lactone Autoinducer Synthase Genes traI and ngrI Increases the Copy Number of the Symbiotic Plasmid in Sinorhizobium fredii NGR234
Source: Front Microbiol. 2016 Nov 18;7:1858. doi: 10.3389/fmicb.2016.01858 (PMC5114275; doi:10.3389/fmicb.2016.01858)

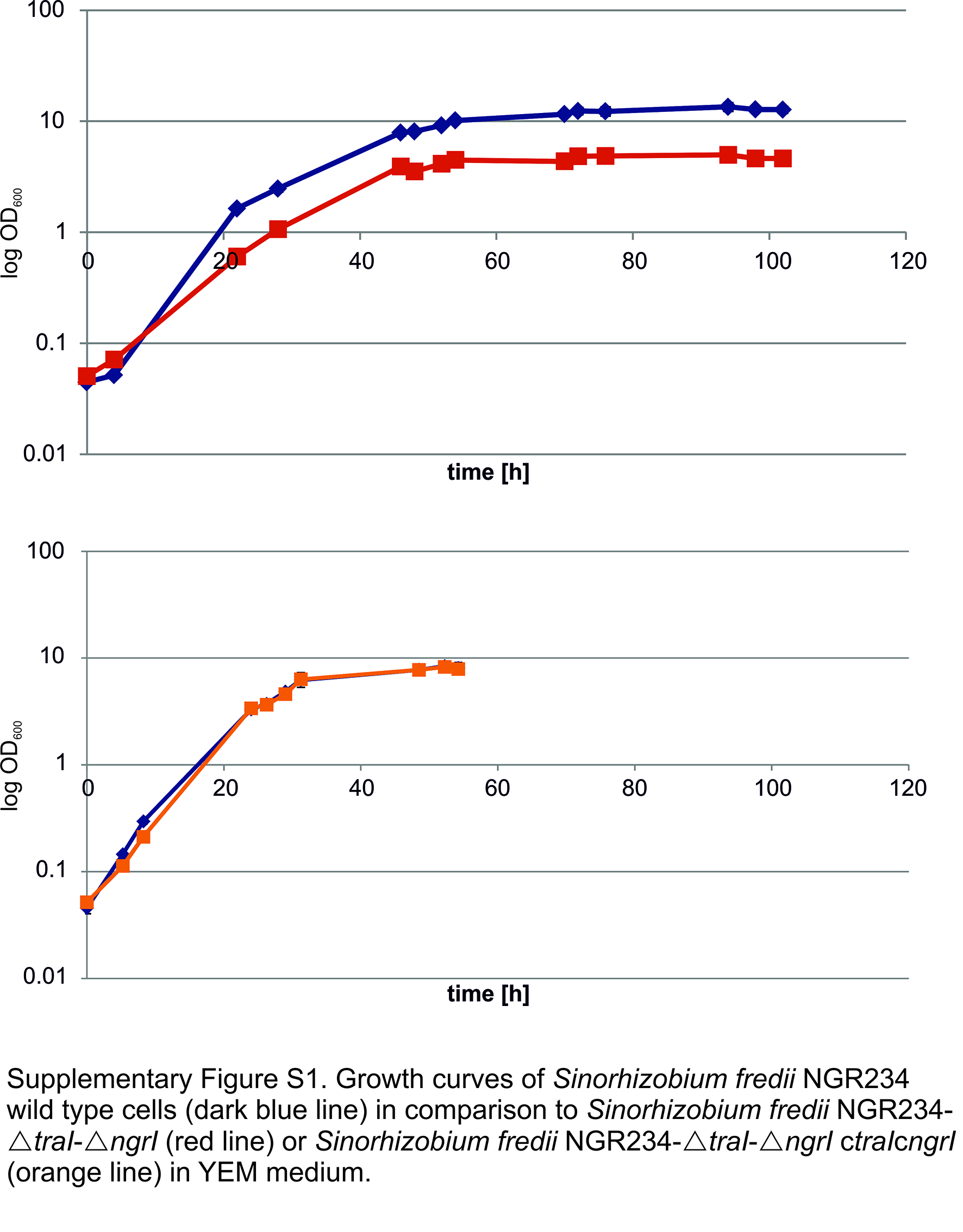

Supplement: Supplementary file 7 [file Image1.TIF]
